# A bone-seeking clone exhibits different biological properties from the ACHN parental human renal cell carcinoma *in vivo* and *in vitro*

**DOI:** 10.3892/or.2011.1572

**Published:** 2011-11-30

**Authors:** JIANG WANG, ANMIN CHEN, CAIHONG YANG, HENG ZENG, JUN QI, FENG-JIN GUO

**Affiliations:** Department of Orthopedics, Tongji Hospital, Tongji Medical College, Huazhong University of Science and Technology, Wuhan 430030, P.R. China

**Keywords:** renal cell carcinoma, bone metastasis, *in vivo* selection, animal model development

## Abstract

Metastatic bone disease caused by renal cell carcinoma (RCC) occurs frequently. Very little is currently known about the mechanism of preferential metastasis of RCC to bone. We hypothesize that RCCs that develop bone metastases have the capacity to facilitate their colonization in bone. To examine this hypothesis, we established bone-seeking (ACHN-BO) clones of the human RCC cell line ACHN by repeated four passages in nude mice and *in vitro* of metastatic cells obtained from bone. These clones were examined for distinguishing biological characteristics and compared with the ACHN parental cells (ACHN-P) *in vivo* and *in vitro*. Our results showed that the ACHN-BO cell line could be successfully obtained by *in vivo* selection through the lateral tail vein. This approach results in the development of multiple osteolytic lesions in the distal femora and proximal tibiae within four weeks after inoculation, with a success rate of 85–100% and no additional comorbidity. ACHN-P cells developed metastases in lung, bone, brain, ovary and adrenal glands. Conversely, ACHN-BO cells exclusively metastasized to bones with larger osteolytic lesions. Compared with the ACHN-P cell line, the proliferation ability in ACHN-BO6 was increased by 9.68 and 6.42%, respectively (P<0.05), while the apoptotic ratio decreased significantly (P<0.05) and cells were blocked in the S phase with suppressed migration and invasion capacities. The ACHN-BO_6_ cell line produced greater amounts of the pro-angiogenic factors VEGF and TGF-β than ACHN-P. Our data suggest that these phenotypic changes allow RCC cells to promote osteoclastic bone resorption, survive and proliferate in bone, which consequently leads to the establishment of bone metastases. This model provides a reliable reproduction of the clinical situation and, therefore, is suitable for designing and evaluating more effective treatments for RCC bone metastasis.

## Introduction

Renal cell carcinoma (RCC) is a common malignancy (1) and >35% of patients dying with RCC have skeletal metastases (2). These skeletal metastases cause devastating complications including intractable bone pain, pathological fractures, and hypercalcemia (2–4). Thus, bone metastasis in RCC is one of the major causes of increased morbidity and eventual mortality and a therapeutic target in RCC patients. Clinically their care is challenging for the orthopaedic surgeon because of the highly lytic, vascular nature of the tumors (5). In order to affect any improvement in survival from this inherently chemo- and radio-resistant disease (6–8), a better understanding of the molecular mechanisms involved in RCC growth in bone is required.

Generally, it has been recognized that cytokine secretion by RCC cells into the local microenvironment modulates host immune response, tumor growth and metastasis (9). RCC are highly vascular tumors, which overproduce angiogenic factors such as vascular endothelial growth factor (VEGF) (9,10). Most cancer cells produce transforming growth factor-β (TGF-β) and a high level of TGF-β secretion is thought to increase the malignant potential of the tumor. Increased plasma levels of TGF-β were described as a tumor marker and prognostic factor in RCC (11). The pro-inflammatory cytokines interleukin-6 (IL-6) and interleukin-8 (IL-8) were also predominantly produced by RCC (12–15). IL-6 in particular is an autocrine growth factor for RCC and seems to be tumor protecting against cytotoxic tumor-infiltrating lymphocytes (11,16–17). The importance of the TGF-α/EGF-R signaling pathway in RCC bone metastasis has been elucidated by Weber *et al* (9).

We reasoned these phenotypic changes might be responsible for the mechanism of preferential metastasis of RCC to bone. To examine this hypothesis, we established bone-seeking (ACHN-BO) clones of the human RCC cell line ACHN by repeated sequential passages in nude mice and *in vitro* of metastatic cells obtained from bone. These clones were examined for distinguishing biological characteristics and compared with the ACHN parental cells (ACHN-P) *in vivo* and *in vitro*. We found that the bone-seeking clone of ACHN cells has several different biological properties from those of the ACHN parental cells. Our data suggest that these phenotypic changes may allow RCC cells to develop and accelerate bone metastases.

## Material and methods

### Cell cultures

ACHN cells, a human renal cancer line, were from China Center of Type Culture Collection (CCTCC), Wuhan (China). ACHN cells were maintained in DMEM/F12 (1:1) medium supplemented with 10% heat-inactivated fetal bovine serum. All cell lines were incubated at 37°C in a humidified atmosphere of 5% CO_2_ in air.

### Animals

For selection *in vivo*, 6- to 8-week-old female nude mice (BALB/c-nu) were obtained from the Experimental Animal Center of Huazhong University of Science and Technology (Wuhan, China). All animals in our study were housed under pathogen-free conditions and maintained according to the guidelines of the Committee on Animals of Huazhong University of Science and Technology.

### In vivo selection of ACHN-BO

Female nude mice (BALB/c-nu, 5-week-old) were anesthetized with 5% Rompun/10% Ketavet in 0.9% NaCl/10 g body weight before injection. We injected 0.1 ml of tumor cells (5×10^5^ cells) into the lateral tail vein of anesthetized BALB/c-nu mice using an insulin syringe as described by Otsuka *et al* (18). Mice with osteolytic lesions in the hind limbs caused by ACHN-P line detected by radiography (osteolytic lesions in hind limbs were monitored every 7 days starting from day 28) were sacrificed and the affected hind limb was separated from the body. Skin and muscles were removed and the hind limb was mashed with a piston through a sieve in a petridish containing 10 ml of 0.9% NaCl medium. Tumor cell suspension was collected from the petridish into a T-75 flask. The next day, cells were washed twice with PBS to wash off mouse bone marrow cells that did not attach to the plate. After 3 weeks, a population of human cancer cells was obtained. These subpopulations (ACHN-BO_1_) were again inoculated into the lateral tail vein of anesthetized female BALB/c-nu. Following four passages of *in vivo* selection a highly bone metastatic cell line, ACHN-BO_4_, was obtained. The mice were sacrificed when they lost >10% of their body weight.

### Radiographic analysis of osteolytic lesions

Osteolytic lesions in hind limbs were monitored every 7 days starting from day 28 using a Digital Faxitron small animal X-ray cabinet (Faxitron X-Ray, Wheeling, IL, USA) at 35 kV tube voltage, 0.3 mA current and 4 sec exposure time. Lesion area in hind limbs was quantified using image analysis software (Analysis Software Imaging System GmbH, Germany).

### Bone histology

For histological examination, hind limbs were removed from mice after being sacrificed, fixed in 4% buffered formalin (Merck & Co. Inc., USA), decalcified in 10% EDTA (Sigma-Aldrich, Munich, Germany), dehydrated and embedded in paraffin. Tissue sections of 4 μm thick were cut and stained with hematoxylin and eosin (H&E) using standard protocols.

### Cell growth assay

To assess possible impacts of *in vivo* selection on ACHN cells malignant biological behaviors. The cells were trypsinized, counted, plated and assayed for cell proliferation, migration and invasion in triplicate experiments by our research group (19). For proliferation assays, cells in the log-growth phase were harvested, suspended at a density of ~1×10^4^ cells/μl and seeded into triplicate wells of 96-well plates at 100 μl/well. After 24 h of culture, 50 μl of 1X MTT was added to each well. The plates were then incubated at 37°C for 4 h. After removal of the supernatants, the precipitates were solubilized in DMSO (150 μl/well) and shaken for 20 min. The absorbances of the wells were measured at a wavelength of 450 nm.

Sterile polycarbonate membrane filters (Corning Inc., New York, NY) with 8-μm pores were coated with 6 μg/ml Matrigel gelatin (BD Co., Franklin Lakes, NJ). The filters were hydrated with 200 μl of serum-free medium at 37°C for 60 min before use. Cells (5×10^4^) were seeded into the top chambers of 6-well plates, and the lower chambers were filled with 500 μl of DMEM/F12 (1:1) medium containing 10% FBS for 24 h. The filters were fixed with 95% alcohol and stained with hematoxylin for 15 min. The cells on the upper surface were gently removed with a cotton swab and the cells on the lower surface of the filters were quantified under a microscope at a magnification of ×400.

For invasion assays, Matrigel-coated sterile 8-μm polyethylene filters were rehydrated as described above. The lower chambers of 24-well plates were filled with 1 ml of DMEM/F12 (1:1) medium containing 10 μg of fibronectin as a chemoattractant and 0.5 ml of serum-free DMEM/F12 (1:1) containing 5×10^4^ ACHN-P(BO) cells was added to the upper chambers for 48 h. Subsequently, the cells were stained with hematoxylin and the numbers of cells that had invaded the filters were recorded.

### Cell apoptosis assay

Cells were harvested using trypsin/EDTA in phosphate-buffered saline (PBS; Biochrom), counted, and collected by centrifugation in PBS. Phosphatidylserine (PS) exposure on the outer leaflet of the plasma membrane was detected using the fluorescent dye Annexin V-FITC Apoptosis Detection kit according to the manufacturer’s instructions. In brief, cells were rinsed with ice-cold PBS and then resuspended in 200 μl of binding buffer. Annexin V stock solution (10 μl) was added to the cells and incubated for 30 min at 4°C. The cells were then further incubated with 5 μl propidium iodide (PI) and were immediately analyzed on a FACSC-LSR (Becton Dickinson) equipped with CellQuest (Becton Dickinson) software; ~5×10^5^ cells were collected in each of the samples and 5×10^4^ cells were analyzed.

### ELISAs to measure production of growth factors and cytokines

The production and secretion of TGF-α, VEGF, bFGF, and IL-6 (Uscn Life Science Inc., Wuhan, China) by the ACHN-P cell line and the highly metastatic subpopulation (ACHN-BO_4_) were determined in cell culture supernatants after 48 h of culture using a quantitative sandwich enzyme immunoassay technique (ELISA) according to manufacturer’s instructions. Triplicates were measured.

### Data analysis

Data are expressed as the means±standard deviations. Statistical analyses were performed using Student’s t-test. Differences were considered to be statistically significant when the P-value was <0.05.

## Results

### The establishment of ACHN-BO cell line

**Figure 1:**
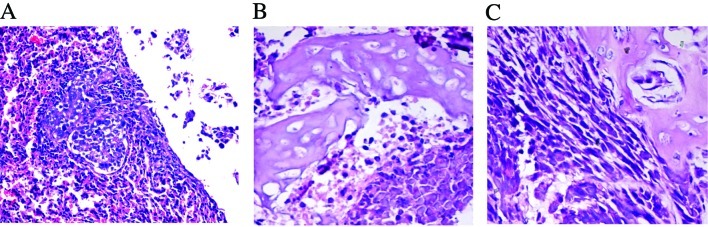
Histological examination of lung (A) and humerus bone metastasis sites (B and C). The sections were thin-cut and stained with hematoxylin and eosin (original magnification, ×200).

In the first cycle of inoculation, 25% (3/12) of the transplanted mice with ACHN-P cells inoculated through the lateral tail vein had tumor clones in bone (the time point was 6 weeks), the remaining 75% (9/12) of the transplanted mice were observed for 4 months and no bone metastasis and no tumor-related death were found. The lung metastasis rates were 100% (11/11) (Fig. 1A). The bone metastasis tumor clones were dissected and used for primary cell culture; these bone metastasis tumor cells were confirmed as ACHN-BO1 cells with histological analysis (Fig. 1B) and chromosomal karyotype analysis (data not shown). In the second cycle of inoculation, ~42% (5/12) of the transplanted mice had bone metastasis. Starting with the third cycle, higher percentage of the bone metastasis in the transplanted mice was observed. All of the transplanted mice had bone metastasis after the fifth inoculation (12/12). Most of the bone metastasis sites were spine and the four limbs. In addition, the tumor cells can also translocate to adrenal glands, sub-mandibular glands, lung and lymph nodes, etc. We named the sixth cycled bone metastasis cells as ACHN-BO_6_ with histological analysis confirmation (Fig. 1C).

### Symptoms of the RCC bone metastasis model resemble clinical setting

**Figure 2:**
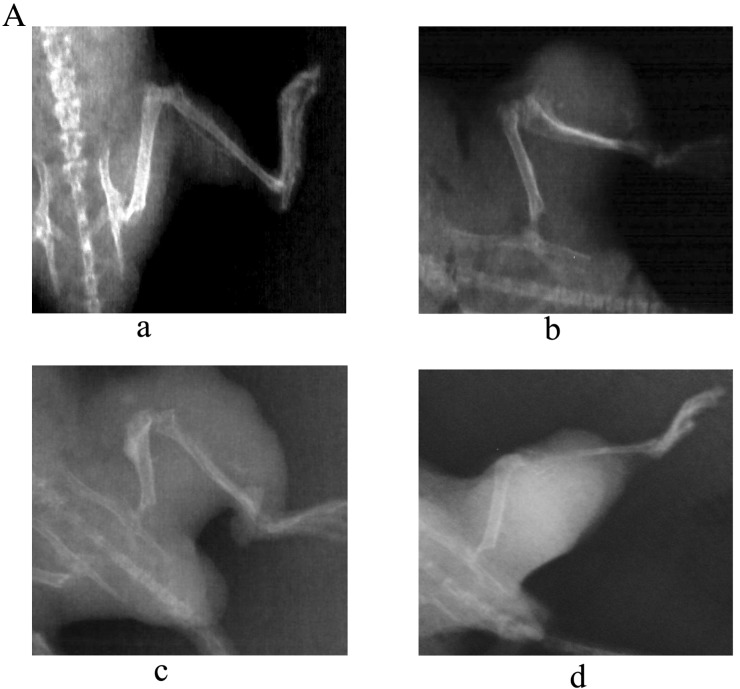

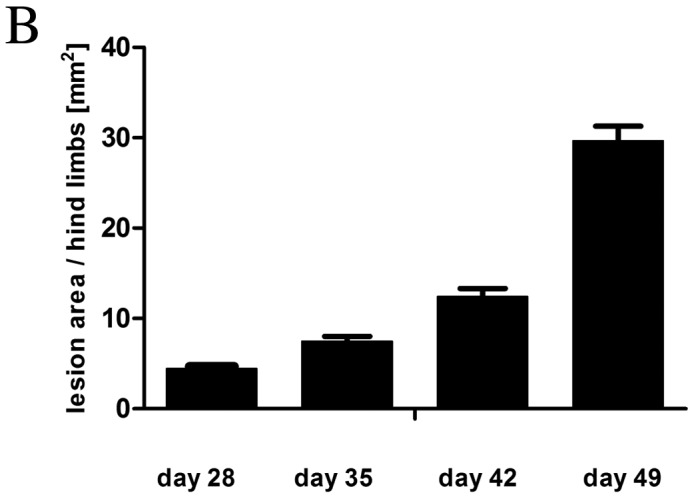
The constinual increase of the osteolytic lesion area in ACHN-BO_6_ bone metastasis model. (A) Representative radiographs showing one hind limb monitored weekly from day 28 until day 49. (B) Mean osteolytic lesion area of hind limbs as measured by radiography (n=8 for each time point).

After ACHN-BO_5_ cell inoculation, all animals developed aggressive osteolytic bone metastasis as monitored by radiography with the endpoint at a mean of 6 weeks. First lesions were observed by radiography 4 weeks after tumor cell inoculation. Fig. 2A shows the time course of the development of osteolytic lesions monitored weekly from day 28 until day 56 by radiography. The determination of mean lesion size from the X-ray images indicated a steady increase of the osteolytic lesion area (Fig. 2B). The observed extensive bone destruction was similar to those noted in clinical settings and mainly occurred in tibia, femur, fibula, forelimbs, spine and sometimes in pelvis.

### The proliferation, migration and invasion of ACHN cells in vitro

**Figure 3:**
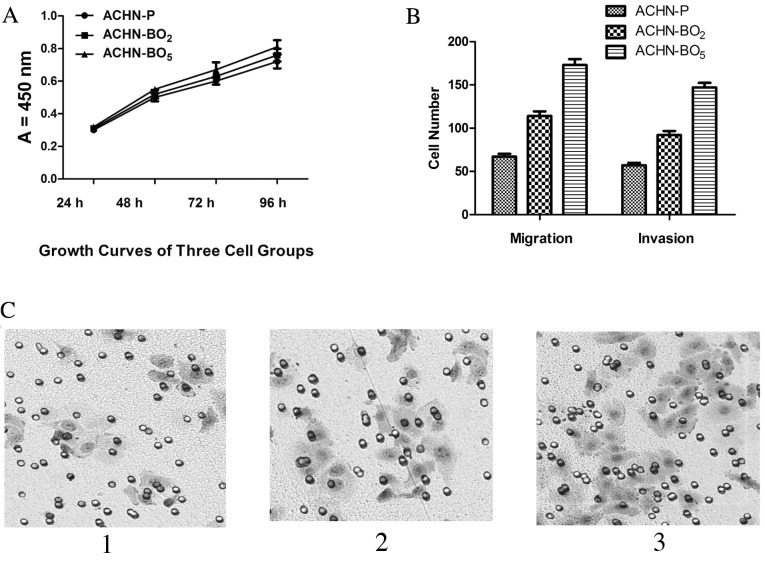
The *in vivo* selection increased proliferation, migration and invasion of ACHN cells. (A) Growth curves of three cell groups evaluated by MTT assays. (B) Comparisons of the cell migration and invasion activities among three cell groups. Compared with ACHN-Ps, the cell migration and invasiveness of ACHN-BO were reduced by 46.71% (ACHN-BO_2_) and 54.24% (ACHN-BO_5_), respectively (P<0.05). (C) The typical invasion photographs of three groups. 1, ACHN-P; 2, ACHN-BO_2_; 3, ACHN-BO_5_.

As shown in Fig. 3A, compared with ACHN-P cell lines; the ACHN-BO exhibited increased cell proliferation by 2.17% (ACHN-BO_2_) and 0.81% (ACHN-BO_5_) for the first 48 h, respectively (P<0.05). After 96 h, the proliferation was significantly increased by 8.97 and 4.72%, respectively (P<0.05). No difference in tumor cell proliferation and *in vivo* tumor growth between the cell lines could be observed (Fig. 3A). We further evaluated whether the selected cell lines altered the motility of ACHN cells across Transwell polycarbonate membranes. As shown in Fig. 3B and C, compared with ACHN-Ps, the cell migration and invasiveness of ACHN-BO were reduced by 46.71% (ACHN-BO_2_) and 54.24% (ACHN-BO_5_), respectively (P<0.05). Taken together, these data suggest that the ACHN-BO cell line significantly inreased the proliferation, migration and invasion of ACHN cells *in vitro*.

### Effects of in vivo selection on ACHN apoptosis

**Figure 4:**
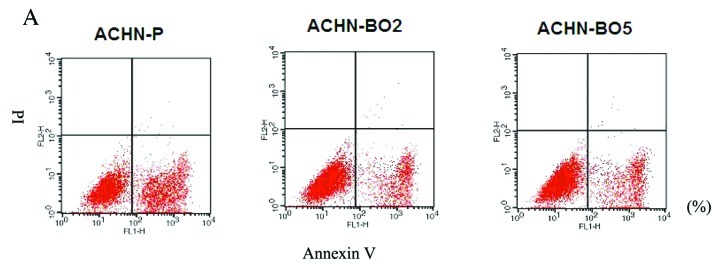

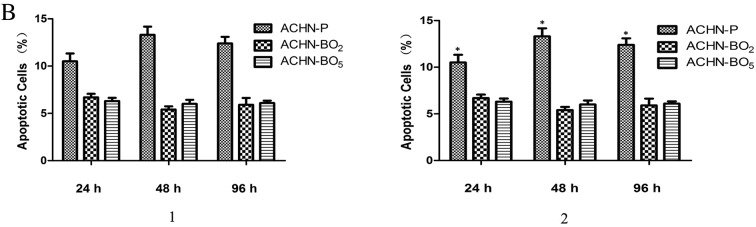
LSS triggers anti-apoptosis in MSCs. MSCs were incubated for 12 and 24 h. (A) Apoptosis was then quantified by FACS analysis after staining with Annexin V and propidine iodide (PI). The Annexin V^+^/PI^−^ (B1) cells are early in the apoptotic process. Viable cells are Annexin V^−^/PI^−^ (B2). Each data point represents mean ± standard deviation of three independent experiments. ^*^P<0.05 vs. static control.

Cell apoptosis was measured using fluorescent dye Annexin V-FITC, which binds to phosphatidylserine residues that are redistributed from the inner to the outer leaflet of the cell membrane as an early event in apoptosis. After loss of membrane integrity, PI can enter the cell and intercalate into DNA. Annexin V^+^/PI^+^ cells were considered necrotic cells, whereas Annexin V^+^/PI^−^ cells were counted as apoptotic cells. Fig. 4 shows the percentages of Annexin V-stained and PI-stained cells of ACHN cell lines. The percentage of Annexin V^+^/PI^−^ cells was reduced to 10.4% compared with the static control value of 13.1% after 24 h and was reduced to 9.7% compared with the static control value of 12.3% after 48 h. With a longer incubation (96 h), the population of Annexin V^+^/PI^+^ cells was much smaller than that of the static control (3.5 vs. 12.7%). These data suggest that the ACHN- BO cell lines had a reduced cell apoptosis compared to ACHN-P cell lines.

### The expression of various growth factors and cytokines

**Figure 5:**
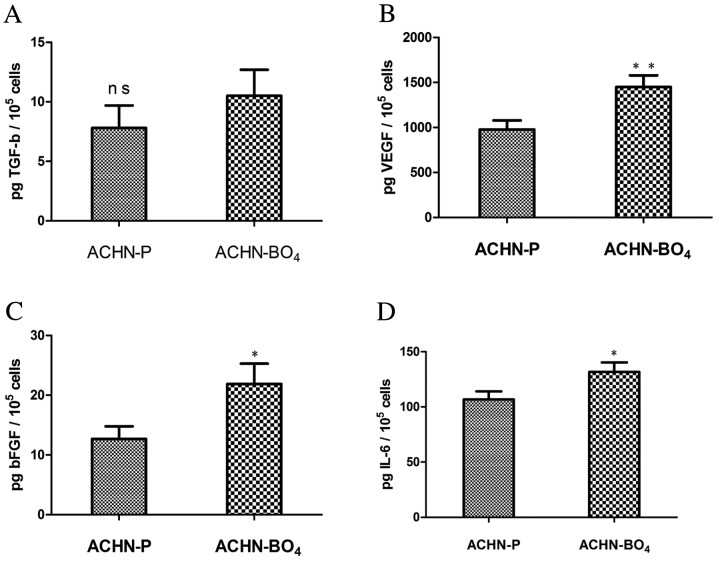
*In vitro* characterization of the *in vivo* selected cell line. Parental and high metastatic cell lines were characterized by quantitative ELISA of (A) TGF-β, (B) VEGF, (C) bFGF and (D) IL-6 *in vitro* (these growth factors and cytokines have been suggested to be important in RCC bone metastasis). Subpopulation ACHN-BO_4_ showed higher secretion of the pro-angiogenic factors VEGF and bFGF, of the osteolytic factor IL-6 compared to the parental cell line. Mean ± SD values shown (^*^P<0.05; ^**^P<0.01; ns, not significant).

The subpopulation with high metastatic potential, ACHN-BO_4_, showed higher or equivalent expression of all analyzed growth factors and cytokines compared with the parental cell line (Fig. 5). The secretion of TGF-α was on the same level in the ACHN-BO_4_ cell line (10.5±2.2 pg TGF-α/10^5^ cells) as in the parental cell line (7.8±1.9 pg TGF-α/10^5^ cells, P=0.148) (Fig. 5A). Pro-angiogenic factors, such as VEGF and bFGF, were significantly induced in the highly metastatic ACHN-BO_4_ *in vivo* selected cell line compared to the parental cell line. VEGF levels were significantly higher in the ACHN-BO_4_ cell line, with a mean of 1450±129 pg VEGF/10^5^ cells compared with 978±101 pg VEGF/10^5^ cells in the parental cell line group (P=0.009). bFGF secretion was ~2-fold higher in the highly metastatic cell line with 21.9±3.4 pg bFGF/10^5^ cells compared with 12.7±2.1 pg bFGF/10^5^ cells in the parental cell line (P=0.026) (Fig. 5B and C). Similarly, the level of IL-6 was also significantly elevated in the highly metastatic cell line (131.7±8.6 pg IL-6/10^5^ cells) compared to parental cell line (106.8±7.2 pg IL-6/10^5^ cells; P=0.019) (Fig. 5D).

## Discussion

In the present study, we validated a model of human RCC bone metastasis as clinically relevant. It rapidly forms growing osteolytic bone metastases after intravenous injection to nude mice and displays distinguishing biological characteristics. We showed that the ACHN-BO cell line has unique properties through the *in vivo* body imaging and subpopulation cell line culture *in vitro*.

Animal models of metastasis have supported drug development and have been useful for identification of metastasis suppressor and promoter genes as novel targets for therapies (20,21). After tail vein injection of ACHN cells, the development of osteolytic lesions were observed after 4 weeks. This nude mouse model is therefore very robust and properly reflects the bone metastatic process observed in the clinic. Tumor cell injection into the left heart ventricle is the standard technique used to induce bone metastasis (22). Intratibial injection of tumor cells is also a challenging procedures to induce bone metastasis (23). Our model requires a more straightforward route of injection. These cells are inoculated intratibially and develop osteolytic lesions similar to those in patients.

TGF-β is among the most abundant growth factor stored in bone (24–26). TGF-β is released continually into the bone marrow cavity in active form as a consequence of bone resorption during physiological bone remodeling (27). Therefore, it is likely that the growth of breast cancer cells metastasized in bone are under the influence of bone derived TGF-β. TGF-β generally is known as a tumor suppressor and inhibits the growth of most cancer cells in culture (28–30). In this study, we have shown that the anchorage-independent growth of the ACHN parental cells and the bone-seeking clone is inhibited significantly by TGF-β. In contrast, TGF-β failed to suppress the anchorage-independent growth of the bone-seeking clone, suggesting that the bone-seeking clone is resistant to the growth inhibitory effect of TGF-β. Our data suggest that this resistance is unlikely because of changes in the expression and activation of TGF-β signaling pathways such as type I and type II receptors, Smad2, Smad3, Smad6, and Sma7. It is possible that other mechanisms that have been shown recently to cause the resistance of cancer cells to the growth inhibitory effect of TGF-β [41–44] play a role (31–33).

As hypervascularity is associated with RCC bone metastasis, we analyzed the pro-angiogenic factors VEGF and bFGF which are reported to be crucial for angiogenesis, proliferation, survival and spread of cancer cells (34). We were able to demonstrate that VEGF and bFGF are induced in the highly metastatic ACHN *in vivo* selected subpopulation compared to the parental ACHN cell line. As reported, the induction of pro-angiogenic factors in the majority of RCC cases is due to the inactivation of the von Hippel-Lindau (VHL) tumor suppressor gene, which is also known to be missing in ACHN cells. The loss of VHL leads to stabilization of hypoxia-inducible factor-1α (HIF-1α) which in turn results in the expression of many proangiogenic factors such as VEGF (35). VEGF is the most important angiogenic cytokine and its overexpression contributes to the typical hypervascular histology of clear cell RCC (36).

Elevated levels of IL-6, an autocrine tumor growth factor produced by RCC cells, correlated with a poor outcome in patients with metastatic RCC (37). Serum levels of IL-6 were detectable in the majority of patients with metastatic renal cell carcinoma and showed a significant correlation to progression-free survival and overall survival (38).

Taken together, the characterization of the ACHN bone metastasis model and the consequent *in vivo* selection approach are the basis for the identification of factors implicated in RCC bone metastasis and provide a possibility to evaluate and improve promising drug candidates. Characteristics of the bone-seeking clones *in vivo* and *in vitro* compared with the ACHN parental cells are distinguish. Our results suggest that these capacities are at least necessary for RCC to develop and accelerate bone metastases. However, it should be noted that these capacities are not specifically attributable to bone metastasis of breast cancer and that additional yet unknown properties are likely involved. In this context, the bone-seeking clone we described herein should provide a useful model for identification of novel genes or molecules that are responsible for bone metastasis in RCC.
